# Autophagic Cell Death and Apoptosis Jointly Mediate Cisatracurium Besylate-Induced Cell Injury

**DOI:** 10.3390/ijms17040515

**Published:** 2016-04-06

**Authors:** Haixia Zhuang, Weili Tian, Wen Li, Xingli Zhang, Jingjing Wang, Yue Yang, Xin Liu, Zhengyuan Xia, Du Feng, Liangqing Zhang

**Affiliations:** 1 Department of Anesthesiology, Guangdong Medical University, Zhanjiang 524001, China; Haixia_Zhuang00@163.com (H.Z.); Jingjing_Wang00@163.com (J.W.); Yue_Yang00@163.com (Y.Y.); kiddodo@gmail.com (X.L.); 2 Guangdong Key Laboratory of Age-Related Cardiac-Cerebral Vascular Disease, Institute of Neurology, Department of Neurology, Affiliated Hospital of Guangdong Medical University, Zhanjiang 524001, China; hanxiaoweili@163.com (W.T.); liwen410@163.com (W.L.); Xingli_Zhang00@163.com (X.Z.); 3 Anesthesiology Research Laboratory, Department of Anesthesiology, University of Hong Kong, Hong Kong, China; zyxia@hkucc.hku.hk; 4 The Department of Developmental Biology, Harvard School of Dental Medicine, Harvard Medical School, Boston, MA 02115, USA

**Keywords:** cisatracurium, autophagosome, apoptosis, mitochondria

## Abstract

Cisatracurium besylate is an ideal non-depolarizing muscle relaxant which is widely used in clinical application. However, some studies have suggested that cisatracurium besylate can affect cell proliferation. Moreover, its specific mechanism of action remains unclear. Here, we found that the number of GFP-LC3 (green fluoresent protein-light chain 3) positive autophagosomes and the rate of mitochondria fracture both increased significantly in drug-treated GFP-LC3 and MitoDsRed stable HeLa cells. Moreover, cisatracurium promoted the co-localization of LC3 and mitochondria and induced formation of autolysosomes. Levels of mitochondrial proteins decreased, which were reversed by the lysosome inhibitor Bafinomycin A1. Similar results with evidence of dose-dependent effects were found in both HeLa and Human Umbilical Vein Endothelial Cells (HUVECs). Cisatracurium lowered HUVEC viability to 0.16 (OD490) at 100 µM and to 0.05 (OD490) after 48 h *in vitro*; it increased the cell death rate to 56% at 100 µM and to 60% after 24 h in a concentration- and time-dependent manner (*p* < 0.01). Cell proliferation decreased significantly by four fold in Atg5 WT (wildtype) MEF (mouse embryonic fibroblast) (*p* < 0.01) but was unaffected in Atg5 KO (Knockout) MEF, even upon treatment with a high dose of cisatracurium. Cisatracurium induced significant increase in cell death of wild-type MEFs even in the presence of the apoptosis inhibitor zVAD. Thus, we conclude that activation of both the autophagic cell death and cell apoptosis pathways contributes to cisatracurium-mediated cell injury.

## 1. Introduction

Cisatracurium besylate has some advantages such as strong and fast efficacy, quick recovery without accumulation, good controllability and independence from liver and kidney [1,2]. Hence, it is an ideal non-depolarizing muscle relaxant [3,4]. Although cisatracurium besylate has some clinical advantages compared to other muscle relaxants, whether this drug has other side effects needs further investigation [5,6]. In 2001, it was reported that cisatracurium besylate could affect proliferation of cells *in vitro* [7]. Another study found that the effect of cisatracurium besylate on cell proliferation could be attributed to apoptosis caused by over-accumulated peroxides [8]. In the present study, we sought to investigate whether long-term use of different doses of cisatracurium besylate will trigger cellular injury and what is the mechanism of action of this drug.

During the early phase of formation of autophagosome (preautophagosome), the Atg12–Atg5-Atg16L complex promotes the expansion of autophagic vacuole from a small vesicle to a semi-circular structure. The direction of membrane extension depends on the position of the Atg5 complex, *i.e.*, the membrane stretches towards the Atg5 complex [9,10]. When the autophagic vacuole with double-membrane structure develops a closed ring shape, the Atg5 complex detaches from the bilayer membrane, leaving behind the membrane-bound light chain 3II (LC3II) in the autophagic vacuole [11]. The combination of Atg5 complex and autophagic vacuole membrane can also promote the transfer and aggregation of microtubule-associated protein 1 light chain 3 (LC3) in autophagic vacuoles [9,10]. Autophagosomes can then fuse with lysosome membranes to form autolysosomes for subsequent degradation of their contents [12,13].

Apoptosis, which is also called Type I programmed cell death (PCD), is characterized morphologically by cell nuclear shrinkage and fragmentation, apoptotic body formation, chromosomal DNA fragmentation, membranolysis and transfer of phosphatidylserine from the cell inner membrane to the outer membrane [14,15,16]. Recent studies have found that besides apoptosis, PCD also includes autophagic cell death (Type II programmed cell death) [17,18,19,20]. It is thought that moderate- to low-level autophagy plays a protective role, but when autophagy occurs at a high level, it causes autophagic cell death [21,22,23,24]. By focusing on the relationship between cisatracurium besylate and autophagic cell death, the present study aimed to explore possible mechanisms behind the inhibitory effect of cisatracurium besylate on proliferation of cultured cells *in vitro*.

## 2. Results

### 2.1. Cisatracurium Besylate Promotes Mitochondrial Breakage, Mitochondrial Protein Degradation and Colocalization of Mitochondria and Autophagosomes

**Figure 1:**
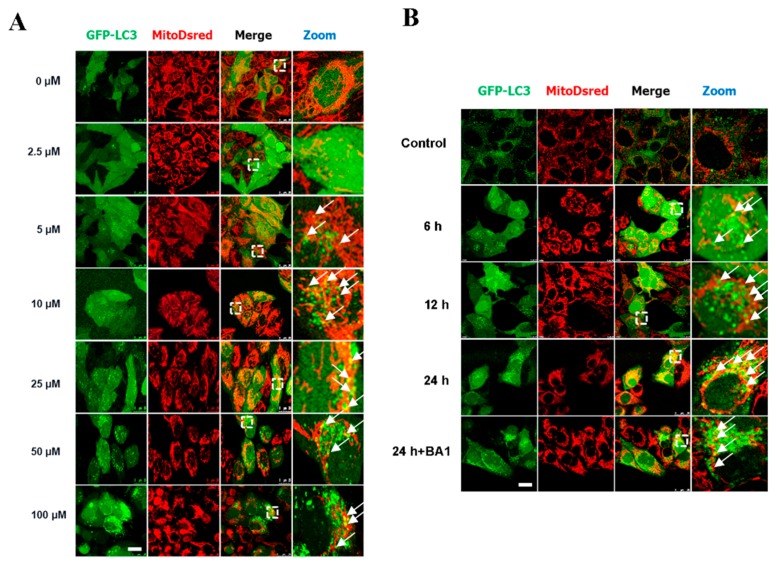

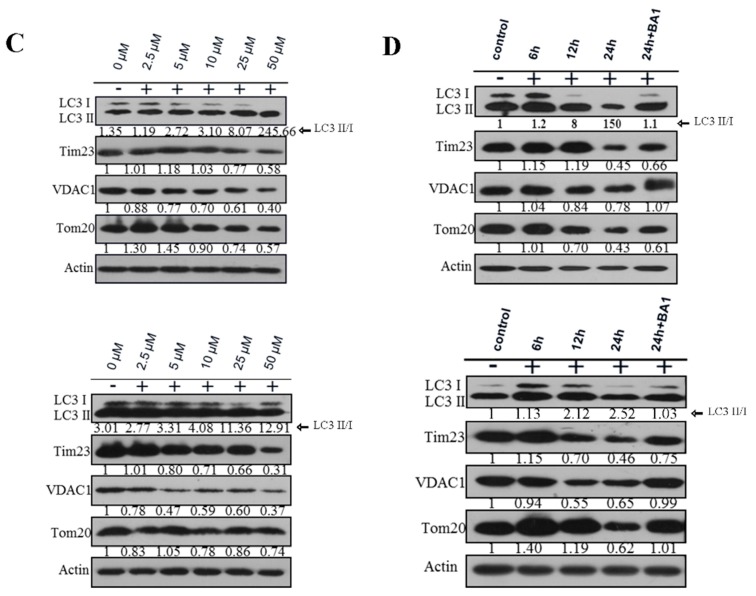
Cisatracurium triggers excessive mitochondrial autophagy in a dose- and time-dependent manner in mammalian cells. (**A**) GFP-LC3 and MitoDsred double fluorescent stable cell line was incubated with cisatracurium (0, 2.5, 5, 10, 25, 50, or 100 µM) for 12 h. GFP-LC3 (green) dot shows autophagosome-like structures. MitoDsred (red) denotes mitochondria. Arrows represent colocalization of LC3-positive autophagosome-like structures with mitochondria. Dotted white frames are enlarged parts of a cell. Bar = 30 µm; (**B**) GFP-LC3 and MitoDsred double fluorescent stable cells were incubated with cisatracurium (5 µM) for 0, 6, 12, or 24 h, in the presence or absence of the lysosome inhibitor Bafinomycin A1 (50 nM, B1793). Arrowheads represent colocalization of LC3-positive autophagosome-like structures with mitochondria. Bar = 30 µm. Arrows indicate colocalized LC3-positive vesicles with mitochondria; (**C**) HeLa (**upper** panel) or HUVEC (**lower** panel) was incubated with cisatracurium (0, 2.5, 5, 10, 25, or 50 µM) for 12 h. Samples were then subjected to immunoblot using antibodies against LC3, TIM23, TOM20, VDAC, or β-actin. Data are presented as representative images from three independent experiments, and the intensity of indicated bands was measured with ImageJ software and normalized by Beta-actin; (**D**) HeLa (**upper** panel) or HUVEC (**lower** panel) was incubated with cisatracurium (5 µM) for 0, 6, 12, or 24 h, in the presence or absence of the lysosome inhibitor Bafinomycin A1 (50 nM, B1793). Samples were then subjected to immunoblot using antibodies against LC3, TIM23, TOM20, VDAC, or β-actin. Data are presented as representative images from three independent experiments, and the intensity of indicated bands was measured with ImageJ software and normalized by Beta-actin.

Selection of cisatracurium besylate concentration was done based on previous studies [2,7,8]. LC3, a protein found on the membrane of autophagosomes, was labeled to track autophagosomes. LC3-I is diffusely distributed in the cytoplasm while LC3-II accumulates on autophagosomes membranes [25]. Upon autophagy induction, cells accumulate large number of autophagosomes. The degree of autophagy can be inferred based on the number of green fluorescent puncta in cell lines stably transfected with GFP-LC3 [26,27,28]. In this study, HeLa cells stably transfected with GFP-LC3 (marks autophagosome-like structures) and MitoDsred (marks mitochondria) were treated with different levels of cisatracurium besylate. Then, LC3 green fluorescent puncta in each cell were counted. Three independent countings were carried out, with at least 100 randomly selected cells used for each counting. With increase in drug concentration, the number of LC3-positive autophagic structures increased in a concentration-dependent manner. Mitochondrial morphology was found to be altered starting at a concentration of 5 µM (Figure 1A). Mitochondria were broken and formed aggregates, and the severity of this phenotype increased with increasing drug concentration. Moreover, colocalization between mitochondria and GFP-LC3 indicated that mitochondrial autophagy was enhanced by the drug. At 100 µM, cell morphology changed from spindle to circular, and mitochondria became more fragmented and aggregated around the nucleus, indicating the occurrence of excessive mitochondrial autophagy (Figure 1A).

Longitudinal observation indicated that cisatracurium besylate could induce mitochondrial autophagy. In addition, autophagosome number and mitochondrial morphology were also found to change over time. HeLa cells stably transfected with GFP-LC3 and MitoDsred were treated with 5 µM cisatracurium besylate at 0, 6, 12, and 24 h. After immobilization and mounting, cells were observed under LCSM and the number of LC3 puncta in each cell was counted. Three independent counts were done, with at least 100 cells randomly selected in each count. As treatment time increased, LC3 aggregates also increased, showing time-dependent (Figure 1B) increase in mitochondrial autophagy. While the control group had largely intact mitochondria, mitochondrial morphology in the treated group had changed after 6 h of treatment. Mitochondria were found to be broken and aggregated in a time-dependent manner (Figure 1B). Moreover, co-localization of GFP-LC3 and mitochondria indicated that mitochondrial autophagy was enhanced with increase in treatment time. Bafilomycin A1 (BA1) is a lysosome inhibitor that can strongly inhibit degradation of autophagosomes [29,30]. The samples were added with BA1 at 8 h before sample collection and observed after 8 h of drug treatment. We found a large increase in the number of autolysosomes, enhanced colocalization of broken mitochondria and autolysosomes, and complete recovery of mitochondrial morphology, indicating that cisatracurium besylate could induce mitochondrial autophagy. When the treatment time was extended to 24 h, cell morphology changed from spindle to circular, and mitochondria were fully fragmented, indicating that mitochondrial autophagy was hyperactivated in those cells (Figure 1B).

To further verify the relationship between cisatracurium besylate and mitochondrial autophagy, we examined change in mitochondrial protein amount and status of transformation from LC3I to LC3II. In mammalian cells, newly synthesized LC3 disperses in the cytoplasm. As autophagy occurs, LC3I is transformed into LC3II, which is inserted into autophagic membranes [31]. Therefore, transformation from LC3I into LC3II, measured as the ratio of LC3II to LC3I, is an indicator of degree of autophagy [32,33]. Accordingly, HeLa cells and HUVECs (Figure 1C,D) were separately treated to examine the expression of mitochondrial proteins, including Tim23 (mitochondrial inner-membrane protein), TOM20 (mitochondrial outer-membrane protein), VDAC (mitochondria intermembrane proteins) and autophagosome protein LC3. Compared with the control group, the ratio of LC3II to LC3I increased gradually in the treatment group with both concentration and time, except for 2.5 µM or 6 h treatment, which led to slight decrease in the ratio (Figure 1C,D). In addition, expression of mitochondrial proteins such as Tim23, VDAC1 and Tom20 were also decreased in a time-dependent manner. After addition of the lysosome inhibitor BA1, expression of mitochondrial proteins returned to levels close to those in the control group. Identical results were observed in two different cell lines, indicating that cisatracurium besylate could easily induce mitochondrial autophagy even at a low concentration (5 µM).

### 2.2. Cisatracurium Besylate Promotes Autolysosome Formation

**Figure 2:**
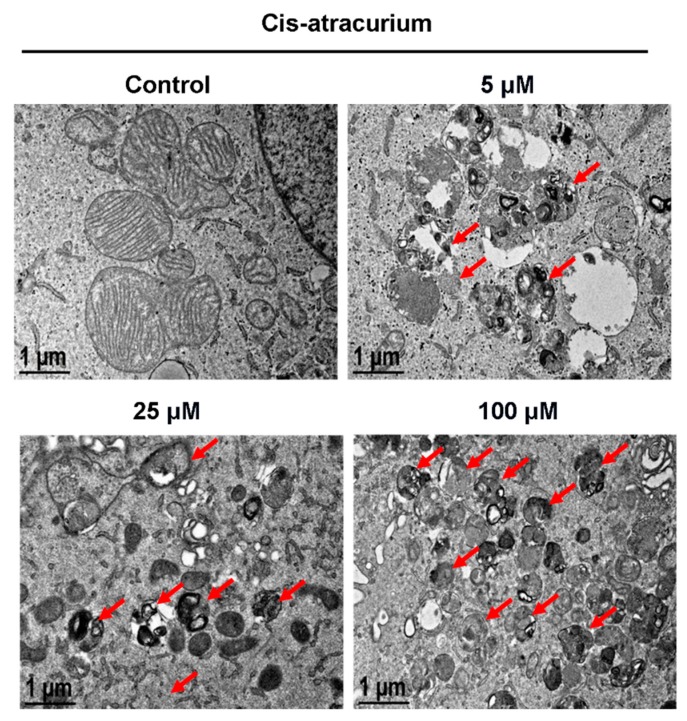
Cisatracurium besylate induces autolysosome formation. HUVECs were incubated with cisatracurium (0, 5, 25, or 100 µM) for 12 h. Samples were analyzed by electron microscopy. Red arrows indicate autolysosomes. The number of the autolysomes increases in a concentration dependent manner.

TEM is commonly used to directly observe the structure of autophagosomes [34,35]. Autophagosomes are double-membrane structures containing undigested cytoplasm or organelles (e.g., injured mitochondria, endoplasmic reticulum fragments). After maturation, they fuse with lysosomes. Autophagosomes show a classical double membrane structure under an electron microscope. At early stages, the electron density of its contents is the same as that of cytoplasm. Hence, it is easy to recognize autophagosomes. After autophagosomes and lysosomes fuse to form autolysosomes, a single-membrane structure that contains cytoplasmic contents at different stages of degradation is formed [36,37,38,39]. The control group showed oval mitochondria with complete crista structure and clear cytoplasm (Figure 2). The drug treatment group showed formation of autolysosomes in a dose-dependent manner (Figure 2). With increase in drug concentration, the number of single-membrane autolysosomes increased. Notably, the degraded products appeared as aggregates of black particles or were amorphous.

### 2.3. Cisatracurium Besylate Inhibits Proliferation of Endothelial Cells in Vitro

**Figure 3:**
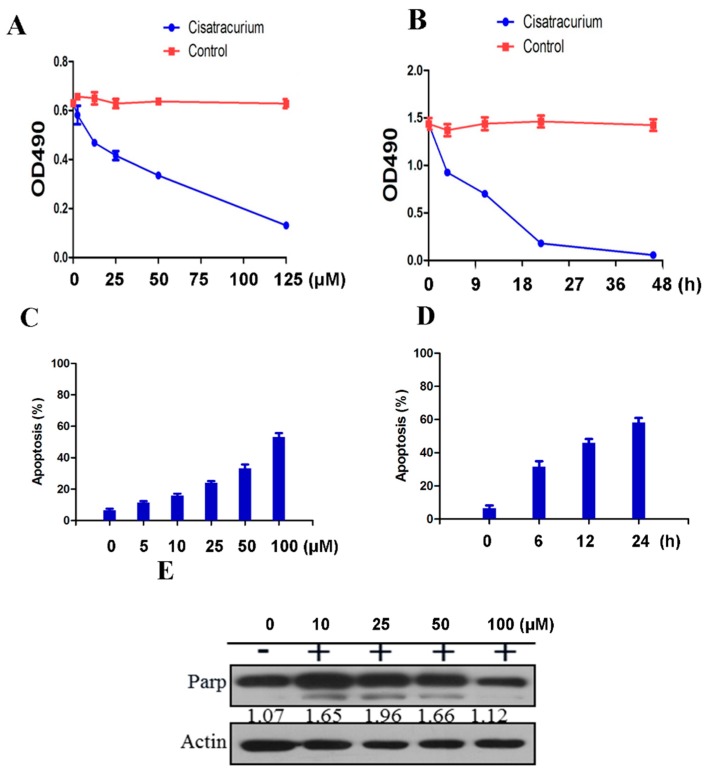
Cisatracurium causes inhibition of cell proliferation and induction of cell death. (**A**) HUVEC was incubated with cisatracurium (0, 5, 10, 25, 50, or 125 µM) for 12 h. Cell viability was analyzed by CCK assay; (**B**) HUVEC was incubated with cisatracurium (10 µM) for 0, 6, 12, 24, or 45 h. Cell viability was analyzed by CCK assay; (**C**) HUVEC was incubated with cisatracurium (0, 5, 10, 25, 50, or 100 µM) for 12 h. Cells were stained by PI and Annexin-5 and then analyzed by flow cytometry to calculate the rate of apoptosis. Data are presented as mean ± SD from three independent experiments. *p* < 0.01; (**D**) HUVEC was incubated with cisatracurium (5 µM) for 0, 6, 12, or 24 h. Cells were stained with PI and Annexin-5, then analyzed by flow cytometry to calculate the rate of apoptosis. Data are presented as mean ± SD from three independent experiments. *p* < 0.01; (**E**) HUVEC was incubated with cisatracurium (0, 10, 25, 50, 100 µM) for 12 h. Samples were then subjected to immunoblot using antibodies against PARP or β-actin. Numbers indicate the normalized optical density ratio of cleaved PARP to β-actin. Data are presented as a representative image from three independent experiments.

Cisatracurium besylate promotes breakage of mitochondria, degradation of mitochondrial proteins, mitochondrial autophagy and drastic changes in cell morphology. Hence, we sought to systematically study the effects of drugs at different concentration and time gradients on endothelial cells *in vitro*. We found that the drug’s inhibiting effect on cell proliferation was concentration-dependent (Figure 3A). Compared with the control group, proliferation of endothelial cells in the drug treatment group showed a significantly linear decreasing trend. The high-concentration treatment group showed the highest decrease in proliferation (*p* < 0.01) (Figure 3A). The inhibitory effect of cisatracurium besylate on the proliferation activity of endothelial cells was also time-dependent (Figure 3B). Compared with the control group, proliferation of endothelial cells in the treatment group showed a significantly linear decreasing trend after treatment at the same concentration (5 µM). The decrease in proliferation was the largest for treatment durations between 6 and 12 h. Over this range of treatment duration, the differences between the control group and the treatment groups as well as those among the treatment groups were all statistically significant (*p* < 0.01) (Figure 3B).

### 2.4. Cisatracurium Besylate Promotes Apoptosis of Endothelial Cells in Vitro

We also used flow cytometry to investigate the extent of damage caused by cisatracurium besylate on endothelial cells *in vitro*. Results showed that apoptosis rate increased in the drug treatment group in a concentration- and time-dependent manner compared with the control group (Figure 3C,D). After 10 µM drug treatment for 24 h, endothelial cell apoptosis rate approached 60%, indicating that long-term application of high-dose cisatracurium besylate did harm endothelial cells *in vitro* (*p* < 0.01) (Figure 3D).

PARP protein is used as a marker of apoptosis in cells. PARP is the main substrate of caspase 3, so PARP cleavage is usually considered an indicator of caspase 3 activation [40,41,42,43]. In the drug treatment group, beginning at cisatracurium besylate concentration of 10 µM, cleavage of PARP proteins could be detected (Figure 3E). Intriguingly, the gray value of cleavage strip did not show prominent gradient variations with increase in concentration, indicating activation of the autophagic cell death pathway (as shown in Figure 1 and Figure 2). At 100 µM, only weak PARP cleavage was detected and both the cleaved and un-cleaved bands showed obvious decrease in intensity, indicating that most endothelial cells were dead at this high concentration (Figure 3E).

### 2.5. Cell Death Induced by Cisatracurium Besylate Depends on Autophagy

**Figure 4:**
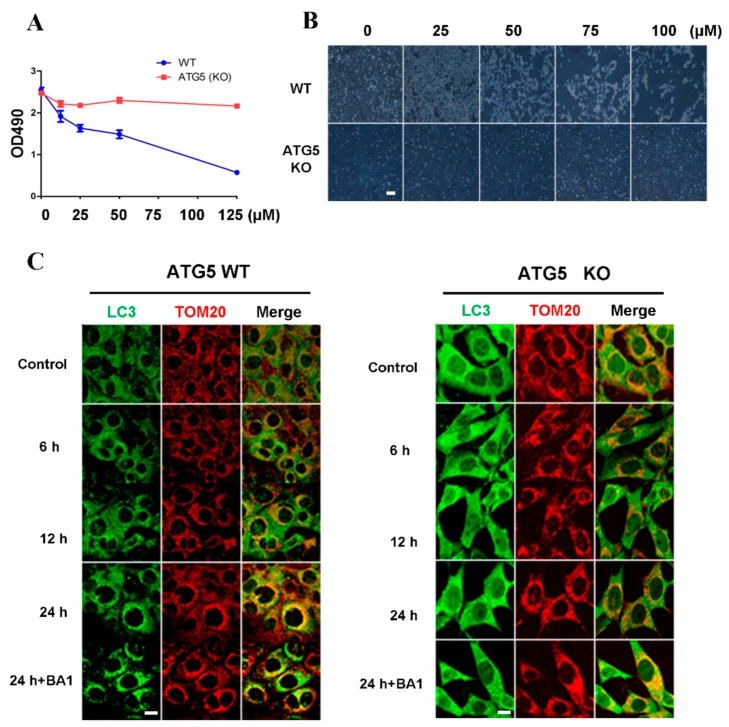

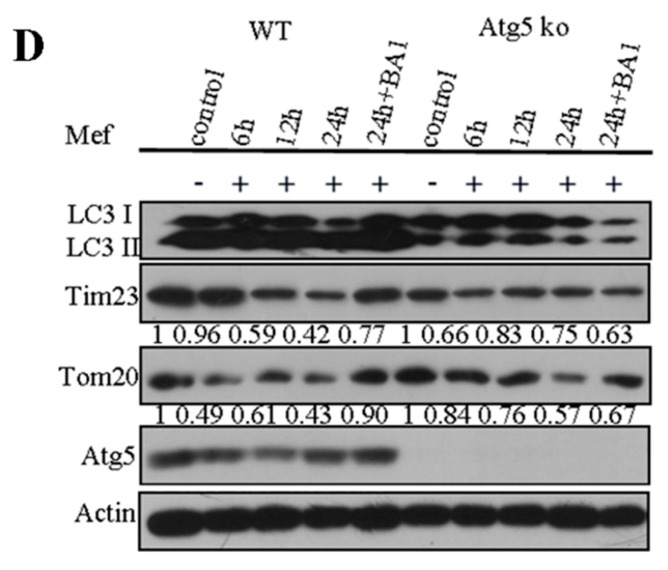
Inhibition of cell proliferation and induction of cell death by cisatracurium is blocked in ATG5-deficient cells. (**A**) WT or ATG5 KO MEFs were incubated with cisatracurium (0, 10, 25, 50, or 125 µM) for 12 h. Cell viability was analyzed by CCK assay; (**B**) WT or ATG5 KO MEFs were incubated with cisatracurium (0, 25, 50, 75, or 100 µM) for 12 h. Cells were observed by light microscopy. Bar = 100 µm; (**C**) WT or ATG5 KO MEFs were incubated with cisatracurium (5 µM) for 0, 6, 12, or 24 h in the presence or absence of Baf A1. Cells were stained by anti-LC3 (green) and anti-TOM20 (red) antibody. Bar = 15 µm; (**D**) WT or ATG5 KO MEFs were incubated with cisatracurium (5 µM) for 0, 6, 12, or 24 h in the presence or absence of Baf A1. Samples were then subjected to immunoblot using antibodies against LC3, Tim23, TOM20, ATG5 or β-actin. Numbers represent the normalized optical density ratio of indicated bands to β-actin. Data are presented as a representative image from three independent experiments, and the intensity of indicated bands was measured with ImageJ software and normalized by Beta-actin.

Since both apoptosis and autophagy occur in cells treated with cisatracurium besylate, we asked if autophagy follows or is independent of apoptosis. In order to address this, we used ATG5 knockout cells to examine the drug effects in these autophagy deficient cells. The hexamer composed of Atg12–Atg5 and Atg16 takes part in the formation of autophagosome precursors [44,45]. Besides, the combination of Atg5 complex and autophagosome membrane can promote the recruitment of LC3-PE to autophagic vacuoles [46]. These facilitate the combination of individual autophagosome precursors to form a complete autophagosome. Some studies have shown significant inhibition of autophagosome formation upon knocking out Atg5 [47,48]. WT MEF cells and Atg5 KO MEF cells were treated with drugs at the range of concentrations described above. Cellular proliferation was detected by the MTT assay method and cell growth was observed under a light microscope. increase in drug concentration, proliferation of WT MEF cells declined linearly (Figure 4A,B). At 10 µM, significant decrease in proliferation was observed. In addition, some cells could not adhere firmly, while some took on irregular shapes, indicating the drug affected cell morphology. In contrast, the drug exerted little effect on proliferation and growth of ATG5 KO MEFs (Figure 4A,B).

In order to further examine whether the effects of cisatracurium besylate on cells rely on mitochondrial autophagy, Atg5 KO MEF cells and WT MEF cells were treated with 10 µM cisatracurium besylate. Observations under a fluorescence microscope revealed that in WT MEF cells, the number of LC3-positive dots increased with time. Moreover, increased colocalization with mitochondria was observed, suggesting occurrence of mitochondrial autophagy (Figure 4C). After drug treatment for 24 h, addition of the lysosome inhibitor BA1 enhanced the colocalization of autolysosome and mitochondrion, suggesting that mitochondrial protein degradation promoted by cisatracurium besylate was mediated via autophagy (Figure 4C). In Atg5 KO MEF cells, neither LC3-positive fluorescent puncta nor changes in mitochondrial morphology were observed over time (Figure 4C).

To further validate these results, WT MEF cells and Atg5 KO MEF cells were treated for various durations, and expression of mitochondrial proteins was examined. In the ATG5 WT group, Tim23 and Tom20 degraded significantly faster than that in the ATG5 KO group after 12 or 24 h incubation in a time-dependent manner. The ratio of LC3II to LC3I was the highest at 24 h. Treatment with the lysosome inhibitor BA1 recovered expression of mitochondrial protein to a level close to that of the control group (Figure 4D). As for ATG5 KO cells, no conversion from LC3I to LC3II was observed. After addition of the lysosome inhibitor, degradation of mitochondrial proteins was not blocked, indicating that cisatracurium besylate-induced mitochondrial turnover occurs through the autophagy pathway (Figure 4D). Thus, our biochemical data and immunofluorescence results both show that apoptosis induced by cisatracurium besylate is mediated via autophagy.

### 2.6. Cisatracurium Besylate Can Promote Cell Death Even in the Presence of the Apoptosis Inhibitor zVAD

As shown in Figure 4, addition of cisatracurium besylate into cells with autophagy deficiency did not induce cell death and barely inhibited cell growth. This indicated that the damage caused by cisatracurium besylate to cells depended on the autophagy pathway. We next asked how cisatracurium besylate would affect cells if they were treated with an apoptosis inhibitor.

**Figure 5:**
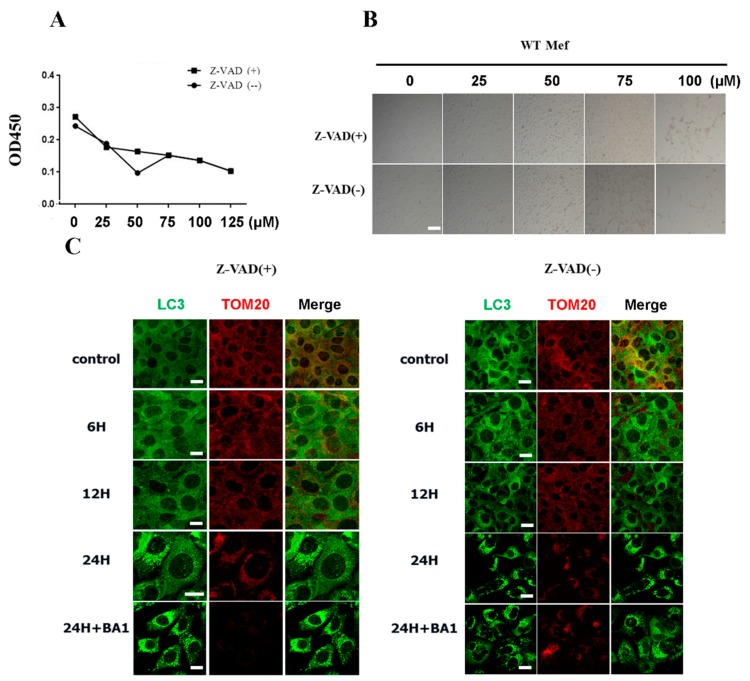
Cisatracurium promotes cell death even in the presence of the apoptosis inhibitor zVAD. (**A**) WT MEFs were incubated with cisatracurium (0, 25, 50, 75, 100, or 125 µM) for 12 h in the presence or absence of apoptosis inhibitor Z-VAD (50 µM). Cell viability was analyzed by CCK8 assay; (**B**) WT MEFs were incubated with cisatracurium (0, 25, 50, 75, or 100 µM) for 12 h in the presence or absence of apoptosis inhibitor Z-VAD. Cells were observed by light microscopy. Bar = 100 µm; (**C**) WT MEFs were incubated with cisatracurium (10 µM) for 0, 6, 12, or 24 h in the presence or absence of Baf A1. Cells were stained by anti-LC3 (green) and anti-TOM20 (red) antibody. Bar = 20 µm.

As shown in Figure 5A, we treated wild-type MEF cells with the apoptosis inhibitor zVAD and added cisatracurium besylate at various concentrations for 12 h. In the presence of 50 µM zVAD, cisatracurium besylate caused linear decline in cell proliferation, and this effect increased with increase in drug concentration. Light microscopic examination revealed significant morphological changes (shrinking and rounding) in cells and decrease in cell number with increase in drug concentration. At 75 µM, most cells died and severe reduction in cell number was observed.

The ability of cisatracurium besylate to induce cell death even when apoptosis is inhibited supports our hypothesis that cisatracurium besylate can injure cells through autophagic cell death. Addition of the apoptosis inhibitor cisatracurium besylate (25 µM) induced autophagy and mitochondrial breakage in wild-type MEFs. Even in the presence of the apoptosis inhibitor zVAD, the number of LC3-positive dots increased significantly with time. In addition, mitochondrial morphology also changed with change in treatment duration. Mitochondria became fragmented gradually and aggregated in the nucleus (Figure 5C). The total number of mitochondria decreased gradually with the most significant change observed after treatment for 24 h. This suggested the occurrence of mitochondrial autophagy in cells treated with cisatracurium besylate (Figure 5C).

## 3. Discussion

Cell death includes apoptosis and non-apoptotic necrotic cell death [49,50]. The latter phenomenon is further divided into autophagic cell death, necrosis and PARP-mediated cell death. Several differences exist between apoptosis and autophagic cell death [49,51,52]. Morphologically, an apoptotic cell has condensed chromosome, fragmented nucleus, plenty of membrane blebs on the plasma membrane, while autophagic cell death is characterized by the accumulation of autophagic lysosomes. Biochemically, apoptosis is mainly mediated by activation of caspase, while autophagic cell death is mediated via cascade reactions of the ATG protein caused by mTOR inhibition. The following criteria are also used to identify autophagic cell death: (1) no involvement of apoptosis; (2) increased autophagy flux; and (3) blockade of cell death upon blocking autophagy [53,54]. A general consensus in the field is that weak or moderate autophagy can protect cells, but excessive autophagy leads to cell death because of excessive digestion of necessary proteins, mitochondria and other essential organelles.

Cisatracurium besylate has various well-known advantages, so it is widely used in clinic. However, some studies have suggested that cisatracurium besylate can affect cell proliferation [7]. Here we found that cisatracurium besylate injured cells via two mechanisms, namely cell apoptosis and autophagic cell death. Due to the simplicity and limitations of previous studies, analysis on the drug’s effect on cell proliferation had been done, but the mechanism of drug action remained unknown. To investigate this, we designed a treatment scheme for HUVEC with several gradient concentrations and durations and examined the effect on cells by multiple approaches. For example, CCK-8 assay was used to determine cellular proliferation, the annexin V/PI double-staining method was utilized to detect apoptosis, Western blot was conducted to determine the level of apoptotic proteins, and immunofluorescence and transmission electron microscopy were adopted to examine changes in mitochondrial autophagy. As in previous studies, we found that inhibition of endothelial cells by cisatracurium besylate depended on drug concentration and treatment duration. More importantly, we found that drug treatment could lead to mitochondrial fragmentation and excessive activation of intracellular autophagy, thereby causing autophagic cell death. In addition, cell death caused by the drug relied on activation of the autophagy pathway, since knocking out core autophagic genes abolished induction of apoptosis and reduction of cell proliferation. Analysis by flow cytometry indicated that in comparison to the control group, sum of the early apoptosis rate (E2) and late apoptosis rate (E4) increased in a concentration- and time-dependent manner. After 6-h treatment with 100 µM drug and 24-h treatment with 10 µM drug, endothelial cell mortality was close to 60%, indicating that high dose and long duration of cisatracurium besylate treatment could damage endothelial cells. In clinical practice, patients need to be continuously infused with cisatracurium besylate for 2–6 h depending on the case [55,56,57,58,59,60]. Occasionally, when the Patient–Ventilator Asynchrony occurs in patients in intensive care with a respiratory machine control, 48 h continuous infusion of the cisatracurium may be applied. During this time period, the drug has continuous effect on the patients’ tissues and cells. Considering our results showing cisatracurium besylate can damage cells, prolonged use of cisatracurium besylate should be done with extreme caution.

Cleavage of PARP is generally used as an indicator of caspase 3 activation and of apoptosis [41,61,62]. In the treatment group, PARP cleavage was detected at cisatracurium besylate of 5 µM. The gray value of cleavage strip showed no gradient variations with increase in drug concentration, *i.e.*, PARP cleavage was not enhanced. In addition, results of PI/Annexin5 double staining revealed that the number of apoptotic cells did not increase further with increase in drug concentration beyond a certain level. This suggested that other non-apoptotic pathways contributed to endothelial cell death induced by cisatracurium besylate. Further investigation is ecessary to determine whether the damage caused by cisatracurium besylate is caused by the metabolite *N*-methyltetrahydropapaverine or excessive accumulation of peroxides (ROS).

The number of intracellular GFP-LC3 fluorescent puncta increased with treatment concentration and duration. Furthermore, fragmentation and aggregation of mitochondria was also observed. Electron microscopic observations revealed that the number of autolysosomes increased significantly and mitochondrial proteins were degraded upon treatment with cisatracurium besylate. Notably, decrease in mitochondrial protein levels was blocked in the presence of a lysosome inhibitor. This suggested the occurrence of mitochondrial autophagy. Furthermore, cisatracurium besylate could not induce apoptosis in cells in which the core autophagic gene ATG5 had been knocked out. Notably, cisatracurium besylate was able to induce autophagic cell death even in the presence of the apoptosis inhibitor zVAD. This is possibly because when the apoptosis pathway is blocked by zVAD, the autophagic cell death pathway would be the major pathway which still works within the cell. Altogether, these results establish autophagic cell death as a new mechanism for endothelial injury caused by cisatracurium besylate.

## 4. Experimental Section

### 4.1. Cell Culture and Antibodies

Human umbilical vein endothelial cell (HUVEC), HeLa, GFP-LC3 and Mito-cherry HeLa and mouse embryonic fibroblast (MEF) cells were grown in Dulbecco’s modified Eagle’s medium (Invitrogen, Carlsbad, CA, USA) supplemented with 10% fetal calf serum (Hyclone, Logan, UT, USA) and 1% penicillin/streptomycin (Beyotime, Shanghai, China, referred to as complete medium) at 37 °C under 5% CO_2_. The following antibodies were used for western blot: anti-TIM23 (BD Biosciences (San Diego, CA, USA) 611222), anti-TOM20 (BD Biosciences, 612278), anti-β-ACTIN (Santa Cruz Biotechnology, Inc. (Santa Cruz, CA, USA) sc-47778), anti-LC3B polyclonal (Sigma-Aldrich (St. Louis, MO, USA), L7543), anti-VDAC1 monoclonal (Abcam (Cambridge, MA, USA), ab14734), HRP Affinipure goat anti-mouse IgG (Earthox (San Francisco, CA, USA), E030110), and HRP Affinipure goat anti-rabbit IgG (Earthox, E030120). Purified mouse anti-CYTOCHROME *c* (BD Biosciences, 556432), anti-LC3 polyclonal (MBL (Woburn, MA, USA), PM036), anti-LC3B monoclonal (MBL, M152–3) antibodies were used for immunostaining.

### 4.2. Cell Viability Assay

The MTT Assay could be either used to measure the cell proliferation rate or the reduction in cell viability. The reduction of tetrazolium salts by NAD(P)H-dependent cellular oxidoreductase enzymes is now widely accepted as a reliable way to examine cell proliferation. HUVECs were seeded into 96-well plates (1 × 10^4^ cells per well). Drug treatments were performed the next day as designed. Then, MTT (50 µL per well) was added to the culture medium and the culture dishes were incubated at 37 °C for 4 h. The media in each well was removed and the insoluble products were dissolved in 150 µL DMSO for 10 min at room temperature. The optical density (O.D.) of each well was measured at 490 nm in a spectrophotometer. The O.D. of the control group represents 100% cell viability.

### 4.3. Cell Apoptosis Assay

Cell apoptosis rate was measured by flow cytometry (Becton Dickinson, Mountain View, CA, USA). The binding of annexin V-fluorescein isothiocyanate (FITC) to phosphatidylserine is an indicator of apoptosis. Cells were double-stained with annexin V-FITC and propidium iodide (PI) according to the manufacturer’s protocols, and cells were then analyzed by FACSan flow cytometer. Annexin V-FITC positive cells reflected the relative proportion of apoptotic cells.

### 4.4. Western Blot Analysis

Expressions of mitophagy and apoptosis related proteins were determined by Western blot. Cells were lysed in RIPA buffer (25 mM Tris HCl, 150 mM NaCl, 1% Nonidet P-40, 0.1% SDS, plus proteinase inhibitors and PMSF; Sigma). Protein concentration was mesured by a Bradford assay kit (Pierce, Rockford, IL, USA), and samples (25 µg) were separated by 4%–12% gradient SDS-PAGE. After transferred, the nitrocellulose membranes were blocked in 5% non-fat milk in 20 mM Tris-HCl, 150 mM NaCl, and 0.05% Tween 20 for 30 min at room temperature, the membranes were then incubated overnight at 4 °C with different primary monoclonal antibodies followed by incubating with the horseradish peroxide-conjugated secondary antibody for 1 h at room temperature and exposed to enhanced chemiluminescence reagents (ECL). Densitometric analysis (ImageJ, NIH, Bethesda, MD, USA) was performed to quantify signal intensity.

### 4.5. Immunofluorescence Microscopy

Cells were grown to 60% confluence on a coverslip. After drug treatment, cells were washed with ice-cold PBS, and fixed with freshly prepared 4% PFA at 37 °C for 20 min. Cells were permeabilized by 0.1% Triton X-100 for 10 min. Cells were incubated with primary antibodies for 60 min, and after washing with PBS, stained with fluorescent dye labeled-secondary antibody for 60 min. Images were captured with a TCS SPF5 II Leica confocal microscope (Leica Microsystems, Wetzlar, Germany).

### 4.6. Electron Microscopy

Electron microscopy was done as previously described [35,63]. Cells were first fixed with 2% glutaraldehyde in sodium cacodylate buffer (pH 7.4) at 37 °C for 2 h, and then dehydrated in a graded ethanol series and embedded. Approximately 75-nm ultrathin sections were mounted on nickel grids. The samples were then stained and visualized using a 120 kV Jeol electron microscope (Peabody, MA, USA) (JEM-1400) at 80 kV. Images were captured using a Gatan-832 digital camera (Pleasanton, CA, USA).

### 4.7. Statistical Analysis

Assays for characterizing cell phenotypes were analyzed by Student’s *t*-test, and correlations between groups were calculated using Pearson’s test. *p* values <0.05 were deemed statistically significant.
